# Maximal strength measurement: A critical evaluation of common methods—a narrative review

**DOI:** 10.3389/fspor.2023.1105201

**Published:** 2023-02-17

**Authors:** Konstantin Warneke, Carl-Maximilian Wagner, Michael Keiner, Martin Hillebrecht, Stephan Schiemann, David George Behm, Sebastian Wallot, Klaus Wirth

**Affiliations:** ^1^ Department for Exercise, Sport and Health, Leuphana University Lüneburg, Lüneburg, Germany; ^2^ School of Human Kinetics and Recreation, Memorial University of Newfoundland, St. Johns, NL, Canada; ^3^ Department of Training Science, German University of Health and Sport, Berlin, Baden-Württemberg, Germany; ^4^ University Sports Center, Carl von Ossietzky University of Oldenburg, Oldenburg, Germany; ^5^ Institute of Psychology, Leuphana University Lüneburg; ^6^ Faculty of Training and Sports Science, University of Applied Science Wiener Neustadt, Vienna, Austria

**Keywords:** maximal strength testing, isometric, dynamic, 1RM, agreement, isometric mid thigh pull, squat, performance testing

## Abstract

Measuring maximal strength (MSt) is a very common performance diagnoses, especially in elite and competitive sports. The most popular procedure in test batteries is to test the one repetition maximum (1RM). Since testing maximum dynamic strength is very time consuming, it often suggested to use isometric testing conditions instead. This suggestion is based on the assumption that the high Pearson correlation coefficients of *r* ≥ 0.7 between isometric and dynamic conditions indicate that both tests would provide similar measures of MSt. However, calculating r provides information about the relationship between two parameters, but does not provide any statement about the agreement or concordance of two testing procedures. Hence, to assess replaceability, the concordance correlation coefficient (*ρ*_c_) and the Bland-Altman analysis including the mean absolute error (MAE) and the mean absolute percentage error (MAPE) seem to be more appropriate. Therefore, an exemplary model based on *r* = 0.55 showed *ρ*_c_ = 0.53, A MAE of 413.58 N and a MAPE = 23.6% with a range of −1,000–800 N within 95% Confidence interval (95%CI), while *r* = 0.7 and 0.92 showed *ρ*_c_ = 0.68 with a MAE = 304.51N/MAPE = 17.4% with a range of −750 N–600 N within a 95% CI and *ρ*_c_ = 0.9 with a MAE = 139.99/MAPE = 7.1% with a range of −200–450 N within a 95% CI, respectively. This model illustrates the limited validity of correlation coefficients to evaluate the replaceability of two testing procedures. Interpretation and classification of *ρ*_c_, MAE and MAPE seem to depend on expected changes of the measured parameter. A MAPE of about 17% between two testing procedures can be assumed to be intolerably high.

## Introduction

Producing high maximal strength is of crucial importance to reach high level performance in many sports such as American football and rugby (1–6), basketball (7–11) handball (12–14), soccer (15–20) swimming (21–23), track and field in sprints, jumps, and throws (24–26) or powerlifting and weightlifting (27–29). Additionally, previous research showed differences regarding strength and speed strength capacities between elite, sub-elite, amateur, and youth athletes (30, 31). Commonly, strength training leads to improved maximal strength and speed strength performance (e.g., sprinting, jumping, rapidly performed directional changes) (24, 32–35).

Especially in elite sports and competitive sports, the effectiveness of training routines is evaluated with performance testing (36) to monitor training progress (24) and, if necessary, adapt training routines. Various methods have been utilized to measure maximal strength using a maximal voluntary contraction (MVC), among which isometric (MVIC) or dynamic testing procedures using the one repetition maximum (1RM) are most common (24, 27, 37).

Advocates of isometric testing highlight the supposed advantages regarding the quantification of various force-time characteristics (1, 38–40), a simple and time efficient administration (1) and a good standardization of test conditions with high test-retest reliability (36, 41). Furthermore, isometric strength tests are considered highly sensitive to changes in strength while possessing minimal coordination requirements, minimal injury risk, and being supposedly less fatiguing than 1RM test protocols (27, 36, 42).

Additionally, measuring force-time characteristics like rate of force development (RFD) or isometric impulse is considered to provide information on various dynamic strength qualities (24, 43). However, dynamic strength measurements using the one repetition maximum (1RM) is stated as the most popular strength assessment method, because no expensive equipment such as a force plates or strain gauges are required (44). Since good reliability can be assumed (Seo et al., 2012), there are a substantial number of studies investigating the 1RM in bench press, back squat or the clean (18, 45–61). To date, there is conflicting evidence about the external validity of various strength testing methods (62), especially when considering the associations between isometric and dynamic performances (43, 63–66). Training and testing specificity (i.e., testing should involve tasks similar to the task or type of training) has been a hallmark of sport and exercise science (67).

Accordingly, a potentially higher transfer of dynamic testing measures towards speed strength performances like sprinting, jumping, rapidly performed directional changes (e.g., agility) seems rational (63, 64, 68). Additionally, 1RM testing provides comparable reliability to isometric testing, with a higher validity to estimate maximal strength capacity (36).

## Problem

Still, based on the supposed advantages of isometric testing conditions and the additional information on force-time characteristics testing has led multiple authors to suggest substituting 1RM testing with isometric testing to monitor athletes' training progress (1, 24) McGuigan et al. (69) state; “Given that the test seems to indicate to a large extent the dynamic performance characteristics of athletes, it may not be necessary to perform 1RM testing on a large number of exercises”. While isometric tests appear to provide valuable information on force-time characteristics, the replaceability of dynamic testing conditions through isometric testing is primarily justified by “high” Pearson's correlation coefficients (r) or intraclass correlation coefficients (ICC). McGuigan et al. (1) also proposed that “Strength and conditioning coaches and other practitioners with access to a force plate can consider using the isometric mid-thigh pull test as a potential alternative to traditional 1RM testing. In recreationally trained subjects, it appears to correlate extremely well with both the 1RM squat and bench press”. Therefore, a high number of studies were found (see Table 1) highlighting the correlation between isometric and dynamic testing conditions. However, to validly claim the potential substitution of dynamic testing conditions for isometric testing within performance diagnostic protocols, a high concordance between methods must be assumed. But none of the studies in the literature calculated concordance correlation coefficients between isometric and dynamic measurements to verify whether one measurement can actually reproduce the results of the other. This is especially of high importance if the replacement of 1RM bench press testing by isometric mid-thigh pull is suggested (69), which seems to be of questionable validity. Hence, the primary aim of this study is to assess the validity of replacing 1RM with isometric testing by comparing Pearson and concordance correlation coefficients. Moreover, to provide more detailed information the mean absolute error (MAE) and mean absolute percentage error (MAPE) will be provided to detect differences between isometric and dynamic testing.

**Table 1:** studies showing significant correlation coefficients between isometric and dynamic strength testing.

Author	*n*	Technique	Technique2	1-RM	1-RMrel
Lower Body (Single Joint)					
Baker (43)	22	Isometric Knee Extension (90° knee angle and 110° hip angle)	(Half) Squat	Pre: 0.575*; Post: 0.57*	–
Boraczynski (70)	25	Isometric Leg Extension (90° knee flexion)	Half Back Squat	0.780*	0.629*
Requena (15)	21	Isometric Knee Extension (single leg; 90° knee angle and 110° hip angle)	Half Squat	0.58*	–
Lower Body (Multi Joint)					
Bartolomei (71)	20	Isometric Midthigh Pull (140° knee angle and 125° hip angle); Isometric Midshin Pull (73.2 ± 6.8 knee angle and 59.8 ± 4.8)	Deadlift	Isometric Midthigh Pull: 0.55*; Isometric Midshin Pull: 0.78*	–
Bazyler (72)	17	Isometric Squat (90° knee angle; 120° knee angle)	Parallel Back Squat; Partial Squat (100° knee flexion)	IPF90°vsParallel: 0.864*; IPF90°vsPartial: 0.705*; IPF120°vsParallel: 0.597*; IPF120°vsPartial: 0.789*	–
Blazevich et al. (73)	14	Isometric Squat (90° knee angle); Isometric Front Hack Squat (110° knee angle and 90° hip angle)	Back Squat (90° knee angle); Front Hack Squat (110° knee flexion)	ISvsS: 0.77*; IFHSvsFHS: 0.76*	–
De Witt et al. (74)	9	Isometric Midthigh Pull (no information on joint angles)	Deadlift	0.88*	–
Dos’Santos et al. (75)	43	Isometric Midthigh Pull (135–145° knee angle and 140–150° hip angle)	Power Clean	0.674*	–
Drake et al. (76)	42	Isometric Squat (90° knee angle)	Back Squat (90° knee angle)	0.688*	0.244
Haff et al. (77)	8	Isometric Midthigh Pull (144 ± 5° knee angle and 145 ± 3° hip angle)	Dynamic Midthigh Pull	0.80*	–
Haff et al. (78)	6	Isometric Midthigh Pull (127–145° knee angle)	Snatch; Clean and Jerk	Snatch: 0.93*; Clean and Jerk: 0.64	–
Markovic & Jaric (79)	159	Isometric Squat (120° knee angle)	Smith Machine Squat (80° knee angle)	0.52	0.38
McGuigan et al. (69)	26	Isometric Midthigh Pull (no information on joint angles)	Half Back Squat	0.97*;	–
McGuigan et al. (80)	8	Isometric Midthigh Pull (130° knee angle)	Parallel Back Squat; Power Clean	Parallel Back Squat: 0.96*; Power Clean: 0.97;	–
Miller et al. (81)	23	Isometric Midthigh Pull (142.91 ± 4.22° knee angle and 140.13 ± 4.77° hip angle)	Hex Bar Deadlift	0.695*	–
Nuzzo et al. (25)	12	Isometric Squat (140° knee angle); Isometric Midthigh Pull (140° knee angle)	Back Squat (70° knee angle); Power Clean	ISqt vs. Back Squat: 0.624*; IMTP vs. Power Clean: 0.740*	ISqt vs. Back Squat: 0.080; IMTP vs. Power Clean: 0.348
Spiteri et al. (7)	12	Isometric Midthigh Pull (140° knee angle and 140° hip angle)	Back Squat (90° knee angle)	–	0.810*
Townsend et al. (82)	23	Isometric Midthigh Pull (self-selected knee and hip angles)	Parallel Front Squat; Hang Clean	Parallel Front Squat: 0.705*; Hang Clean: 0.89*	–
Wang et al. (3)	15	Isometric Midthigh Pull (self-selected knee and hip angles)	Parallel Back Squat	0.866*	–
Young & Bilby (83)	18	Isometric Squat (100° knee angle)	Universal Squat Machine (90° knee angle)	0.71*	0.53*
Beckham et al. (27)	12	Isometric Midthigh Pull	Snatch; Clean and jerk	Snatch: 0.830*; Clean and jerk: 0.838*;	Snatch: 0.808*; Clean and jerk: 0.788*
Upper Body (Multi Joint)					
Murphy et al. (66)	13	Isometric Bench Press (90° elbow angle)	Bench Press	0.78*	–
Murphy et al. (65)	13	Isometric Bench Press (90° and 120° elbow angle)	Bench Press	90°: 0.78*;	–
Baker et al. (43)	22	Isometric Bench Press (unilateral; initial position of the concentric phase of the bench press)	Bench Press	Pre: 0.568*; Post: 0.614*	–
Lum & Aziz (84)		Prone bench pull test (90° and 120° elbow angles)	Prone bench pull	90° *r* = 0.833, 120° *r* = 0.858	–

1RM, one repetition maximum; 1RMrel, one repetition maximum relative to body weight; ISqt, isometric squat; Sq, Squat; IPF, isometric peak force; IFHS, isometric front hack squat; pre, correlations in pre-test; post, correlation in post-test.

* Significant correlation.

**Table 2:** Correlation coefficients in combination with concordance and variance analysis including MAE, MAPE and maximal percentage error.

Figure	Pearson correlation coefficient (r)	Intraclass correlation coefficient (ICC)	Concordance correlation coefficient (*ρ*_c*)*_	Mean absolute error (MAE) in N	Mean absolute percentage error (MAPE) in %	Maximal percentage error
Figure 1	0.92 (0.903–0.939)	0.914 (0.892–0.932)	0.9 (0.88-9.92)	139.99	7.12	27.25%
Figure 2	0.7 (0.632–0.754)	0.696 (0.630–0.753)	0.68 (0.61–0.74)	304.51	17.36	57.24%
Figure 3	0.55 (0.464–0.630)	0.55 (0.461–0.630)	0.53	413,58	23,59	67.42%

**Table 3:** Examples of angle specificity of squat force output

Study	Example 1 deep or parallel squat	Example 2 half squat	Example 3 quarter squat
Bazyler et al. (107)	Parallel 148.2 ± 23.4 kg	Partial squat (100°knee angle): 224 ± 40.1 kg	
Keiner et al. (55)	Parallel (60°knee angle): 75.4 ± 20.8 kg (1.1 ± 0.2 relative to body weight)	Half squat (90°knee angle): 109.2 ± 27 kg (1.5 ± 2.3 relative to body weight)	Quarter squat (120°knee angle): 155.4 ± 28.6 kg (2.1 ± 0.3 relative to body weight) in the
Kubo et al. (123)	Full squat 78.8 ± 14.6 kg	Half squat 95.0 ± 16 kg	
Hartmann et al. (124)	Deep squat 1.15 ± 0.17 relative to body weight		Quarter squat 2.96 ± 0.57 relative to body weight.
Lum & Joseph (125) isometric squat	90° knee angle 1543.9 ± 318.6 N	Isometric squat 120° knee angle 1899.0 ± 459.2 N	
Bartolomei et al. (71)	Mid-thigh pull MVC 2725.3 ± 536.6 N	Mid-shin pull 1967.2 ± 293.3 N	

## Critical evaluation of commonly performed concordance determination

Investigating the concordance between two measurement devices is a well-known problem in medicine (85–88). In the literature, just stating correlation coefficients seems to be insufficient, since agreement and correlations are two different concepts (89). Since they can be assumed to be conceptually different, using the method to calculate correlations must be considered inappropriate or inadequate to investigate agreement “Agreement is a concept that is closely related to, but fundamentally different from and often confused with correlation.” (89). To investigate the agreement of measurements, a high reproducibility is required. The assumption that both measurement devices measure the same parameter needs to be validated with the deviation between the two devices determined to estimate the concordance or lack of concordance. Pearson correlation coefficients only describe the relationship between two parameters but do not provide any information about the agreement between two testing conditions (85, 88). The concordance correlation coefficient can be used instead, assuming a 45° line crossing the origin of the coordinative system and determining the concordance to the regression line of the Pearson correlation (88, 90–92). Furthermore, assuming two testing methods would measure the same parameter, there should be very little variance between them. To illustrate the level of variance between two testing conditions, Bland-Altman Analysis is recommended (85–87, 89, 90, 93). Since the Bland-Altman Plot can be used only for qualitative and visual analysis of variance, the MAE and MAPE are used for quantitative calculation error between both testing conditions. The MAE is stated as a measuring of errors between paired observations evaluating the same parameter (94, 95), while the MAPE (96–98) can be seen as an expression of accuracy, providing quantitative information about the deviation between two measuring techniques. Therefore, both parameters can be stated to investigate the difference between a measured and predicted parameter and were further used to validate testing batteries (99, 100). In other research fields, such as pharmacology and medicine, using the concordance correlation coefficient and Bland-Altman analysis is very common to evaluate the accuracy, validity, and reliability of blood pressure or heart rate devices (91, 92, 101). Since there are no common classifications in high, moderate, and low concordance as found with Pearson correlations (e.g., *r* = 0.2–0.5 small, *r* > 0.5–0.7 moderate, *r* > 0.7 high correlation) (102), it is suggested to classify those effects dependent on content. Assuming moderate increases in maximal strength of for example, 10%–12% within six weeks of training (103), 13.3% within 10 months in elite soccer player (U19) (35) or 12 ± 2%–19 ± 2% in elite cross-country skiers following 12 weeks of strength training (104), the possibility of replacing 1RM testing with isometric testing requires a high concordance with very little variance, rived from the MAPE. If two tests have a concordance variation of 6% but the training-induced change was 12%–13%, there would be an approximate 50% difference in the strength estimate between the two measures, which would not provide acceptable sensitivity or validity. Accordingly, Dominguez-Jiménez et al. (105) described poor concordance in blood sample measuring devices with concordance correlation coefficients of 0.68–0.8, suggesting that the border for poor agreement with concordance correlation coefficients would be <0.9 (106).

Consequently, assuming high correlation coefficient, the calculation of the concordance correlation coefficient and Bland-Altman analysis including MAPE, and MAE were carried out assuming *r* = 0.9 and *r* = 0.7. Data were compiled and added from previous investigations. The MAE was determined using, *n* = number of participants, *x_i_*_ _= the isometric strength value, and *y_i_*_ _= dynamic strength value

$$MAE=\frac{1}{n}*\overset{n}{\underset{i=1}{\sum }}\left|x_{i}-y_{i}\right|,$$

and the mean absolute percentage error (MAPE) using

$$MAPE=\frac{100\%}{n}*\overset{n}{\underset{i=1}{\sum }}\left|\frac{x_{i}-y_{i}}{x_{i}}\right|.$$

**Figure 1:**
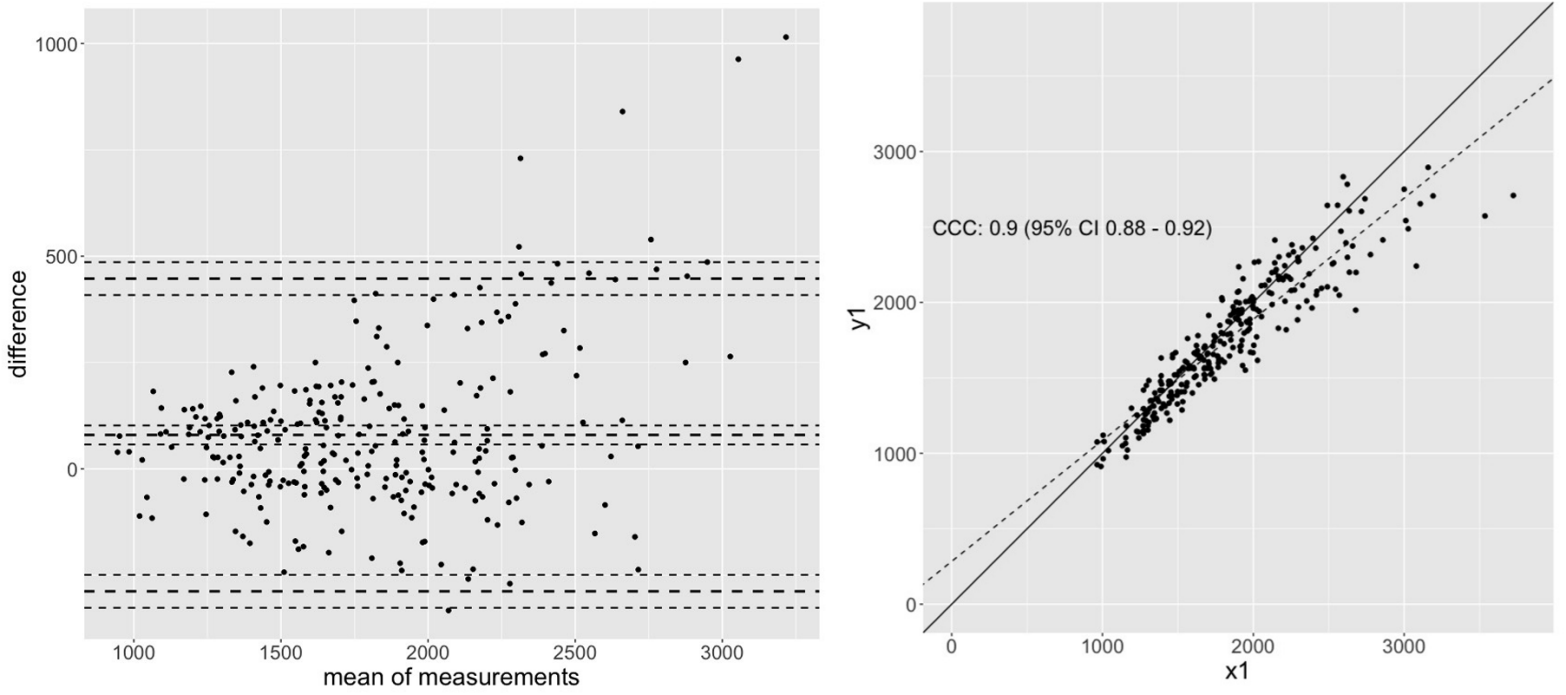
An exemplary dataset to calculate the concordance correlation coefficient (CCC) of 0.9 with *n* = 273 showing high Pearson correlation with *r* = 0.92, representing magnitude of correlation usually found between dynamic and isometric testing.

**Figure 2:**
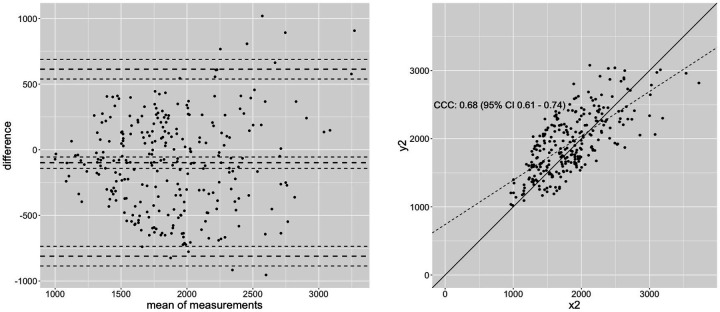
An exemplary dataset to calculate the concordance correlation coefficient (CCC) of 0.68 with *n* = 273 showing high Pearson correlation with *r* = 0.7, representing magnitude of correlation usually found between dynamic and isometric testing.

Results of this exemplary calculation shows that correlations stated as high (*r* ≥ 0.7), which are partially higher than the stated correlations in literature with *r* = 0.52–0.97 (7, 48, 69, 79, 80, 107) seem not to be sufficient to evaluate the replaceability of dynamic testing conditions with isometric testing. Expecting increases between 10%–19% with a strength training program of 6–24 weeks, a MAPE between isometric and dynamic testing of 7%–17% seems to be intolerably high, considering scientific quality criteria (see Table 2). Therefore, both measurement techniques seem to be reliable and valid to estimate specific metrics of maximal strength capacity, however, it must be assumed that they estimate the maximal strength capacity in different ways, providing different results. The rationale to replace 1RM tests with isometric testing conditions (69, 69) must therefore be rejected. The Bland-Altman analysis in Figure 1 showing a variation of values from −200–450 N for *r* = 0.92, in Figure 2 with −750 N–600 N for *r* = 0.7 and −1,000–800 N for *r* = 0.55 within the 95% CI, underpin the assumption of substantially different strength value estimates by isometric vs. dynamic testing conditions. Although Pearson correlation coefficients and ICC values examine the relationship between two parameters, using these common correlation classifications to examine the replaceability of two measurements must be described as a misinterpretation of statistics and should be avoided. In accordance with Cohen (1988), classification of effect sizes should be considered in the light of content. Accordingly, stated substantial higher borders made by Cataldi et al. (106) considering a cutoff of poor (<0.90), moderate (0.90–0.95), substantial (0.95–0.99), and almost perfect (>0.99) seems more appropriate because of reduced errors between the different measurements.

**Figure 3:**
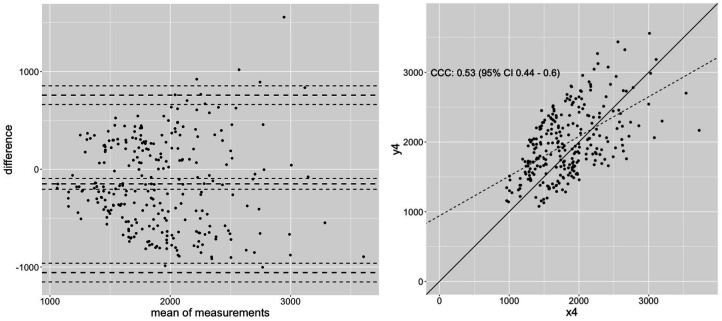
An exemplary dataset to calculate the concordance correlation coefficient (CCC) of 0.53 with *n* = 273 showing moderate Pearson correlation with *r* = 0.55, representing magnitude of correlation usually found between dynamic and isometric testing.

There are few studies that include a concordance analysis to investigate minimizing differences in maximal strength testing batteries. Warneke et al. (108) examined the plantar flexors, showing moderate to high correlation coefficients of 0.63–0.77 leading to *ρ*_c_*_ _*= 0.62–0.77 Accordingly, Wagner et al. (109) pointed out that there was only little agreement between isometric and dynamic squat, determining correlations of *τ* = 0.54, MAE of 2080.87 N and MAPE of 67.4%. However, correlated maximal strength values were assessed using different joint angles, so the influence of contraction type could not be excluded. No further investigations could be detected using concordance analyses to assess the agreement between two testing conditions.

To conclude, if the expected change in maximal strength due to the intervention is smaller than the mean (percentage) error between two testing methods, it cannot be assumed that both testing method measure the same parameter and a replacement of one of both testing methods should be avoided. Therefore, the objective of an investigation and the chosen procedure to evaluate strength capacity should be selected carefully, as it can be assumed that 1RM and isometric testing conditions will not measure the same parameter. There are some hypotheses explaining differences in strength values dependent on measurement procedure.

## Explanation of high variation of correlation coefficients and limited agreement between isometric and dynamic testing procedure

From a physical and mechanical point of view, force is defined as F = m * a, so force capability can be described as the ability of the body to accelerate a mass. Maximal strength in a dynamic strength measurement is only assumed to be maximal if the gravitational force acting on the resistance and the force output exerted on the resistance by the individual are equal, so no movement or acceleration of the mass (resistance) would be present. With isometric force measurements, Newton's third principle [for every action (force) in nature there is an equal and opposite reaction] is used to measure the opposing force to an insurmountable resistance. Since performing a one repetition maximum involves moving a surmountable resistance through a range of motion, it is not the same as assessing maximal strength/MVC, leading to the assumption that the 1RM performance would be lower than MVIC. Furthermore, when performing a 1RM, once the initial sticking point is surpassed, the force to move the weight decreases due to inertia (110). In contrast, there are limitations of isometric testing conditions, described in the following.

## Angle specificity in isometric testing conditions

In science and diagnostics, there are high demands on standardization to ensure equal testing conditions and exclude external factors influencing the results. Angle specificity of maximal isometric strength produces different maximal strength values when performed at different joint angles (44, 62, 71, 111, 112). Angle specific differences in maximal strength were reported for the squat (44, 113–116), bench press (65), plantar flexion (115) and deadlift/mid-thigh pull (81). It seems that strength capacity using isometric squat and leg press increases with increasing knee joint (44, 113–116). Examples are stated in Table 3.

The 1RM bench press, back squat and clean are the most common methods of assessing maximum strength in athletes (116–120). Using dynamic testing conditions, standardization is mostly performed for range of motion (69). The squat ROM varies between different studies (mostly 70–110°) (Table 1), (36, 44, 73, 121, 122).

Using different joint angles to standardize movements is of questionable validity as similar levels of flexibility and anthropometrics would be assumed. Participants lacking flexibility could reach maximal muscle length in a smaller joint angle compared to flexibility trained participants. Consequently, assuming a muscle length-maximal strength relationship with highest strength capacity in the “mid-range of motion” (126), standardization in joint angles lead to differences in starting muscle length, if participants demonstrate heterogeneity in flexibility. Furthermore, there are angle dependent differences in EMG- activity, contributing to differences in strength performance (127). Obviously, joint angle dependency for MVIC values leads to a joint angle dependency for correlations between isometric and dynamic MVC testing. Accordingly, Bazyler et al. (107) reported an angle specific high correlation (*r* = 0.79–0.86) in maximal isometric strength with 1RM in the squat indicating that “these findings demonstrate a degree of joint angle specificity to dynamic tasks for rapid and peak isometric force production” (107). However, obviously, there are numerous other factors influencing the force output. The aforementioned difficulties with standardization of testing range of motion and angles with indivuduals with varying levels of flexibility would be problematic not only for closed chain activities (e.g., squat, cleans, deadlifts) but also open kinetic chain exercises such as found with machines for knee extension (quadriceps) and flexion (hamstrings) or elbow flexion (biceps brachii) and extension (triceps brachii). Furthermore, compared to these uni-articular (e.g, knee extensions, bicep curls and others) resistance exercises, the complexity, co-ordination, balance, and stability associated with multi-joint movements will influence the force production leading to higher standardization problems.

## Familiarization with testing conditions

Another possible explanation is a lack of familiarization to isometric testing conditions (23, 76) due to structural, neural, and biomechanical differences within isometric and dynamic testing conditions associated with the distinct movement patterns and contraction modes (63, 64, 128). Accordingly, Baker et al. (43) suggested that isometric and dynamic muscle actions must be understood as different physiological phenomenon as motor unit recruitment and rate coding (firing frequency) may differ between both contraction forms. Authors pointed out that three familiarization sessions or a large number of trials (129) were required to get a high stability and reliability for peak force measurement. Palmer et al. (130) reported the relatively high coefficients of variation of 6.6%–19.4% for isometric squat strength were dependent on the knee angle. These high coefficients of variation may be the result of learning to contract under isometric conditions. Unfamiliar testing conditions can influence test quality criteria, consequently, reliability of isometric testing is not always reached (131). Since it can be assumed that most athletes are familiar with dynamic conditions because of daily use in training context, it can be hypothesized that for most athletes there is habituation regarding unfamiliar testing conditions. Lum et al. (24) point out that many studies investigating relationships between dynamic and isometric conditions do not provide any information about the number of familiarization sessions prior to isometric testing. This may explain the nearly perfect correlations shown by McGuigan et al. (1) when testing wrestlers, where a high proportion of daily training involves isometric work. Taken together, the range of correlation (*r* = 0.35–0.99) (25, 80) can be attributed to several limitations in standardization and familiarization of participants. The familiarization of testing conditions and contraction velocity specificity (67) might influence the differences in correlations between testing conditions in different sports such as soccer (18–20), basketball (8, 132) and weightlifting (133–134). Therefore, it could be hypothesized that the type of contraction used in daily training routines would influence the force output in isometric and dynamic testing, and therefore the resulting correlations between both contraction types. However, the sports-dependency regarding the force output of isometric vs. dynamic testing conditions requires further research.

## Relevance for the testing practice

Several factors influencing the estimation of maximal strength can lead to significant errors dependent on testing conditions in cross sectional study designs. Since high specificity in training regimes can be assumed (67) a question arises about the impact on results of longitudinal testing designs. Accordingly, using isometric testing conditions, Yahata et al. (115) showed significant increases in MVIC using an extended muscle length in response to long-term stretch training. As it can be assumed that the training routine took place with longer muscle length, training adaptations and strength changes were also specific to training conditions. However, comparing isometric and dynamic testing conditions, significant differences in response to training stimuli would be expected. Warneke et al., (135) showed significant increases in strength capacity under isometric as well as dynamic conditions using six weeks of daily stretch training in the calf muscles. However, under isometric testing conditions there was a significant increase of 16.8%, while 1RM testing showed significant increases of about 25.1%. Furthermore, in 1RM testing a significant contralateral force transfer was present (+11.4%), which was not significant under isometric conditions (+1.4%). Wirth (136) investigated the effects of different weekly training frequencies on maximal dynamic and isometric maximal strength with the biceps brachii muscles. While dynamic testing conditions showed significant increases in 5 of 6 training groups, only one group showed significant increases in MVIC.

Consequently, if Yahata and colleagues (115) would test MVIC exclusively using small joint angles or Wirth (136) tested only MVIC, both studies would underestimate effects of the training routine because of inappropriate testing conditions. Furthermore, Warneke et al. (135) were not able to show a significant contralateral force transfer using daily stretch training, if following the advice to replace 1RM testing by isometric testing. Therefore, the different tests should not be replaceable, but supplement one testing condition with the other. Thus, both testing conditions only estimate MSt capacity, since in both procedures, limitations avoid a “real” maximal force output. Therefore, it is strongly recommended to keep in mind high specificity of testing and training conditions considering the physiological background of each when figuring out the research hypothesis and the following testing protocol.

## Conclusion

The use of correlation coefficients to justify the replaceability of 1RM testing with isometric testing seems invalid, since the MAPE and MAE between both measurement procedures is intolerably high, even when high correlation coefficients with high sample sizes were used. Investigating the agreement between two measurement conditions requires further analytic approaches, such as concordance- and Bland-Altman analyses with classification of MAPE and MAE values. Investigations considering adequate analyses are very rare in exercise science. Results showing that both 1RM and MVIC present a different estimation of the maximal strength capacity of the participant. Therefore, assuming there are equivalent measures between dynamic performance and isometric testing conditions (24, 84) should be questioned. This estimation can be assumed to be influenced by very different factors such as tested muscle lengths in isometric testing, complexity of the movement in dynamic testing as well as familiarization with the testing conditions considering the type of contractions used in daily training practice.

## Practical applications

Using maximal strength tests in practice—performance diagnostics in sports or pre-post-test designs in scientific studies—authors should consider limitations which should be minimized. Since a higher transfer of 1RM to sport specific movements can be assumed and most athletes using dynamic movements in their daily training routines, a higher application of dynamic testing protocols can be hypothesized in field tests (64, 109) “From this, it could be recommended to use dynamic strength testing and avoid isometric strength testing, if the athletes training routine includes only low level of isometric contractions, and vice versa.” (109). However, under laboratory conditions and dependent on the research questions, isometric procedures can also be useful, especially because of time-saving aspects. Whether, and to what extent isometric testing conditions can considered safe might depend on the tested movement. Safety benefits of the isometric squat, pushing the spine against an unyielding resistance may be questionable, while in other movements such as the plantar flexion, the isometric measurement seems to be a safe testing condition. High test specificity (often involves dynamic testing) and relevant physiological issues (often necessitates isometric testing) should be included in the testing design to answer research questions adequately. To avoid missing potential training effects, authors and coaches should be aware of the underlying physiological mechanisms of their training to determine target-oriented testing programs, otherwise there are too many parameters (e.g., different joint angles) to consider, if all possible movement executions should be tested.
